# The CSAW Study (Can Shoulder Arthroscopy Work?) – a placebo-controlled surgical intervention trial assessing the clinical and cost effectiveness of arthroscopic subacromial decompression for shoulder pain: study protocol for a randomised controlled trial

**DOI:** 10.1186/s13063-015-0725-y

**Published:** 2015-05-09

**Authors:** David Beard, Jonathan Rees, Ines Rombach, Cushla Cooper, Jonathan Cook, Naomi Merritt, Alastair Gray, Stephen Gwilym, Andrew Judge, Julian Savulescu, Jane Moser, Jenny Donovan, Marcus Jepson, Caroline Wilson, Irene Tracey, Karolina Wartolowska, Benjamin Dean, Andrew Carr, the CSAW Study Group

**Affiliations:** Nuffield Department of Orthopaedics, Rheumatology and Musculoskeletal Sciences, The Botnar Research Centre, University of Oxford, Headington, Oxford, OX3 7LD UK; Nuffield Department of Population Health, University of Oxford, Old Road Campus, Roosevelt Drive, Oxford, OX3 7LF UK; Institute for Science and Ethics, University of Oxford, Suite 8, Littlegate House, 16/17 St Ebbe’s Street, Oxford, OX1 1PT UK; School of Social and Community Medicine, University of Bristol, Canynge Hall, 39 Whatley Road, Bristol, BS8 2PS UK; Nuffield Department of Clinical Neurosciences, John Radcliffe Hospital, Headley Way, Headington, Oxford, OX3 9DU UK

**Keywords:** Acromion, Arthroscopy, Placebo, Randomise, Sham, Shoulder, Trial, Waiting list

## Abstract

**Background:**

Arthroscopic subacromial decompression (ASAD) is a commonly performed surgical intervention for shoulder pain. The rationale is that removal of a bony acromial spur relieves symptoms by decompressing rotator cuff tendons passing through the subacromial space. However, the efficacy of this procedure is uncertain. The objective of this trial was to compare the efficacy and cost-effectiveness of ASAD in patients with subacromial pain using appropriate control groups, including placebo intervention.

**Methods/Design:**

The trial is a three-group, parallel design, pragmatic, randomised controlled study. The intervention content for each group (ASAD, active monitoring with specialist reassessment (AMSR) and investigational shoulder arthroscopy only (AO)) enables assessment of (1) the efficacy of the surgery against no surgery; (2) the need for a specific component of the surgery—namely, removal of the bony spur; and (3) quantification of the placebo effect. Concealed allocation was performed using a 1:1:1 randomisation ratio and using age, sex, baseline Oxford Shoulder Score (OSS) and centre as minimisation criteria. The primary outcome measure is the OSS at 6 months post randomisation. A total of 300 patients recruited over 24 months from a minimum of 14 UK shoulder units over 24 months were required to detect a difference of 4.5 points on the OSS (standard deviation, 9) with 90% power and to allow for 15% loss to follow-up. Secondary outcomes include cost-effectiveness, pain, complications and patient satisfaction. A substantial qualitative research component is included. The primary analysis will be conducted on the modified intention-to-treat analysis. Sensitivity analysis will be used to assess the robustness of the results with regard to the underlying assumptions about missing data using multiple imputation.

**Discussion:**

This trial uses an innovative design to account for the known placebo effects of surgery, but it also will delineate the mechanism for any benefit from surgery. The investigational AO group is considered a placebo intervention (not sham surgery), as it includes all components of subacromial decompression except the critical surgical element. Some discussion is also dedicated to the challenges of conducting placebo surgery trials.

**Trial registrations:**

UK Clinical Research Network UKCRN12104. Registered 22 May 2012.

International Standard Randomised Controlled Trial ISRCTN33864128. Registered 22 June 2012.

ClinicalTrials.gov NCT01623011. Registered 15 June 2012.

**Electronic supplementary material:**

The online version of this article (doi:10.1186/s13063-015-0725-y) contains supplementary material, which is available to authorized users.

## Background

The prevalence of shoulder complaints in the United Kingdom is estimated to be 14%, with 1% to 2% of adults consulting their general practitioner annually regarding new-onset shoulder pain [1]. Rotator cuff pathology, including rotator cuff tears and subacromial pain, reportedly accounts for up to 70% of shoulder pain problems [2]. Other common causes of shoulder pain include frozen shoulder, calcific tendonitis and osteoarthritis (OA).

Painful shoulders pose a substantial socioeconomic burden. Disability of the shoulder can impair ability to work or perform household tasks [3] and can result in time off work [4]. Shoulder problems account for 2.4% of all general practitioner consultations in the United Kingdom [5] and 4.5 million visits to physicians annually in the United States [6]. With the exception of fractures and traumatic rotator cuff tears, most shoulder pain problems are treated initially with conservative care. In some patients with persistent symptoms, surgery may be required. More than 300,000 surgical repairs for rotator cuff pathologies are performed annually in the United States, and the annual financial burden of shoulder pain management in the United States has been estimated to be US$3 billion [7].

Subacromial pain is the most frequent cause of shoulder problems in the general population. An anatomic aetiology has been proposed whereby mechanical contact occurs between the rotator cuff tendons and the overlying acromion and coracohumeral ligament. Subacromial pain and rotator cuff tears are associated with progressive change in the shape of the acromion, with ‘spurs’ forming at its anteroinferior margin. Evidence suggests spurs develop which narrow the subacromial space, thereby making physical contact more likely, particularly in certain positions of the arm (known as a ‘painful arc’), and resulting in inflammation [8,9]. This is sometimes referred to as *impingement*. However, this term suggests a definitive mechanism of the pain, and conflicting theories indicate that such mechanisms do not fully explain all shoulder pain. For the purposes of the present study, we will continue to refer to this as *subacromial pain*.

A high proportion of patients with subacromial pain respond to conservative treatment alone [10]. The most frequent indications for surgery are persistent and severe pain combined with functional restrictions that are resistant to conservative measures. Some reports suggest that surgery may be no more effective than physiotherapy in the relief of pain after variable attempts at conservative care. Contradicting these reports are cohort studies describing good outcome after surgery [11,12]. The most common surgical intervention for subacromial pain is a subacromial decompression (SAD), which can be performed using an arthroscopic SAD (ASAD) approach. An assessment of the cost of treatment of impingement also suggested that the addition of surgery, in comparison to exercise treatment alone, is not cost-effective [13].

Further research should aim to identify whether removal of subacromial spurs associated bursa and release of the coracoacromial ligament is beneficial, which would thereby help to rationalise surgical treatment.

### Rotator cuff tears

One possible cause of subacromial pain is a rotator cuff tear. The term *rotator cuff tear* refers to structural failure and tissue disruption in at least one of the four muscles and tendons that form the rotator cuff. Any tear that involves rotator cuff disruption that does not extend all the way through the tendon is termed a *partial thickness tear* (PTT). PTTs are more prevalent than full-thickness tears [14]. Information is lacking regarding the risk of progression of PTTs to full-thickness tears, although it is suggested that lesions involving more than 50% of the thickness of the cuff are at risk of progression in the long term [15]. The management of PTTs is controversial, and patients with PTTs have commonly been treated conservatively. If the symptoms fail to resolve with conservative treatment, then ASAD might be beneficial. Favourable results have been reported following debridement of PTT in association with ASAD [16].

### Subacromial decompression surgery

It is widely assumed that arthroscopic SAD (ASAD) has some therapeutic benefit. This assumption is based on the belief that much of the pain and symptoms of subacromial pain is due to mechanical contact between the upper surface of the rotator cuff tendons and the undersurface of the acromion. This pain is also thought to be associated with inflammation of the intervening subacromial bursa.

ASAD has been performed for the treatment of subacromial pain and rotator cuff disease for the last 35 years. The number of ASADs performed by orthopaedic surgeons has increased significantly over time, a fact made remarkable by the absence of any compelling or concrete evidence in support of the procedure. A 254% increase between 1996 and 2006 (from 29.9 to 102.2 per 100,000 people per year) in use of the procedure in New York State was reported, with only a 74% increase in orthopaedic surgery overall and a 475% increase (from 3.3 to 19.0 per 100,000 people per year) in use of the procedure in Olmsted County, Minnesota, in the 25 years leading up to 2005 [17,18]. Although both studies showed a significant increase in use of the procedure, the New York State surgeons performed over five times as many decompressions per capita as their colleagues in Rochester, Minnesota.

The introduction of less invasive arthroscopic techniques accounts for some of the overall increased rate of surgery, but it does not explain the geographic variation. Patient and disease characteristics have not changed over time, and there is a growing concern that this procedure is being overused. Observational studies of ASAD show positive results in terms of pain reduction and functional outcome, with high patient satisfaction rates. However, equally good outcomes have been noted in two studies in which researchers followed patients who had arthroscopic rotator cuff debridement or open rotator cuff repair in the absence of SAD [19,20]. Furthermore, comparative studies of ASAD versus nonoperative treatment options, such as physiotherapy, have not shown any significant difference in outcomes between the two treatment modalities [21-26]. There are a number of studies in which investigators have tried to assess the effectiveness of SAD in comparison with a control. In an important, recently reported randomised controlled trial (RCT), ASAD plus subacromial bursectomy was compared with bursectomy alone, and the researchers reported no significant difference in clinical outcomes between the two groups. This finding suggests that removing acromial spurs might not be necessary [27].

Such studies support the theory that undergoing a surgical intervention for subacromial pain carries a significant placebo effect and that removal of the subacromial spur of bone may not be necessary. Unfortunately, no RCTs have been performed on patients with subacromial pain to show that ASAD is more effective than simply inserting the arthroscope, as per investigative arthroscopy, or doing nothing at all (no treatment). The UK Database of Uncertainties about the Effects of Treatment confirms the lack of evidence. It highlights the low level of evidence available and the high susceptibility of bias which exists in some of the publications on this topic. Therefore, we remain ignorant of the mode of action of any potential therapeutic effect. All three options have yet to be tested satisfactorily [28-33].

## Methods/Design

### Ethical approval

UK National Health Service (NHS) ethical approval was obtained on 2 February 2012 from the National Research Ethics Service (NRES) South Central–Oxford B Research Ethics Committee (REC) (12/SC/0028). Local NHS research and development approvals have been gained for each recruiting centre. The study has been accepted on to the UK Clinical Research Network portfolio.

### Concept

The trial is a three-group, parallel, pragmatic RCT. By performing three two-way comparisons, we will test differences between groups. Patients will be randomised in a 1:1:1 ratio. Blinding will be performed for both patients and post intervention assessors in each of the surgical interventions groups (ASAD and arthroscopy-only (AO) groups). Blinding of the assessors will be possible only for the active monitoring with specialist reassessment (AMSR) group.

The first null hypothesis for this trial is that there is no difference between ASAD and AMSR in patients for whom conservative care has failed. AMSR involves no surgical treatment, and the patients who receive it will be considered the control group in this comparison. If this null hypothesis is rejected, with ASAD showing benefit, then ASAD may be an effective treatment. If this null hypothesis cannot be rejected, then ASAD may be ineffective, and its continued use as a surgical intervention should be questioned.

However, there is also a need to understand how potential treatment differences are achieved, notably whether removal of the bone spur is necessary during surgery or whether undergoing arthroscopic shoulder surgery alone (without bony excision) produces similar effects. Further comparisons, including the use of a placebo surgical intervention, will provide this information.

A second null hypothesis is that there is no difference between ASAD and AO in patients for whom conservative care has failed. If this null hypothesis cannot be rejected (or in the unlikely event of the AO group showing greater benefit), then the study may indicate that the proposed critical surgical element—removal of the bone spur—is not necessary to achieve a treatment effect. Should the hypothesis be rejected (with ASAD having greater benefit), then bone removal can be deemed necessary and can be identified as a critical element of surgery. The mechanism will be delineated, and a greater understanding of the mechanical requirements of the surgery will be obtained. Furthermore, this comparison introduces placebo as an important factor in the design, with the specific relevance and influence of placebo detailed subsequently.

The third and final comparison will be between the AO and AMSR groups, in which the value of surgery will be quantified, be it in the form of placebo effect or true physiological effects. An inability to reject the null hypothesis for AO versus AMSR will suggest that arthroscopy, without bone removal, has no significant clinical benefit. In contrast, rejection of the null hypothesis in favour of AO over AMSR will indicate that some form of surgery, be it placebo effect or not, can produce useful treatment effects compared with simple monitoring.

As this design is innovative and involves placebo aspects, more detailed reasoning for the comparisons is given below.

The first comparison testing for treatment efficacy of ASAD against no treatment is straightforward. In the trial, we will examine standard care (theoretically the treatment of choice—ASAD) against no surgical intervention, and the trial will provide a clear answer to the question of treatment efficacy of routinely performed ASAD.

To identify which aspect of the surgery produces benefit, there is a need to take account of any placebo effects of the operation. This requires the introduction of a sham or placebo component. Unlike other well-known placebo or sham surgery trials, such as the Moseley *et al*. knee trial for OA [28],(in which arthroscopic lavage/debridement was shown to be no better than an entirely sham operative procedure, the placebo for the Can Shoulder Arthroscopy Work? (CSAW) Study is not sham surgery, but rather a placebo intervention. A placebo surgical intervention is a surgical treatment which contains all components of the surgical procedure under investigation in an identical sequence, but with the active or critical part of the procedure omitted. We have defined this as the *critical surgical element* [29]. In our CSAW example, the AO group will have surgery that contains all components of the standard ASAD procedure, but without removal of the bony spur.

The third comparison between AO and active monitoring is less consequential in terms of changing practice, but it does provide further insight into how shoulder surgery for acromial pain achieves any benefit. Any observed differences found in favour of AO over AMSR (the control) would enable the improvement to be attributed to either (1) the more general effects of arthroscopic shoulder surgery (but not bone removal) or (2) the placebo effect associated with undergoing surgery.

In summary of the concept, the proposed critical surgical element in this trial is removal of the bone spur–associated bursal tissue and release of the coracoacromial ligament. The design of the trial not only tests the efficacy of surgery but also, by accounting for the likely placebo effect associated with undergoing surgery, allows the true therapeutic mechanism of the surgery to be identified.

### Qualitative study

Recruitment in many RCTs is challenging because of difficulties in explaining and justifying to patients the concepts inherent in the design (such as randomisation and uncertainty) and because many contemporary RCTs require comparison of very different interventions (such as surgical and nonsurgical treatments) [30]. There is a dearth of robust evidence about effective strategies to improve recruitment into RCTs [31]. However, qualitative research has been used to understand recruitment into specific RCTs [32-36] and has been shown to improve recruitment in some cases [37,38]. A qualitative recruitment investigation (QRI) will be integrated into the CSAW Study to identify sources of recruitment difficulties and to suggest changes to improve levels of informed consent and randomisation.

The QRI will monitor recruitment into the CSAW Study in participating centres to obtain information on the characteristics of recruitment into a mixed intervention surgical trial (both surgical and nonsurgical treatment groups). It will also identify sources of recruitment difficulties (refer to Additional file 1). Anonymised feedback and suggestions will be provided to the Chief Investigator (CI) and the Trial Management Group (TMG) to improve the recruitment process, rates of randomisation and informed consent.

### Objectives

The primary objective is to compare ASAD against a nonsurgery arm (the AMSR group) to assess efficacy. In addition, both ASAD surgery and AMSR will be separately compared with investigational shoulder arthroscopy (the AO group) to delineate the mechanism of any benefit. The primary outcome measure is the Oxford Shoulder Score (OSS) at 6 months.

Secondary objectives include the comparison of the three treatment arms in the CSAW Study with regards to other variables—namely, cost-effectiveness, self-reported outcomes (OSS) at 12 months, shoulder function, range of motion, pain, complications, treatment expectations and patient satisfaction, anxiety and depression.

### Number of centres

Participants will be recruited at NHS hospitals throughout the United Kingdom. An initial minimal set of centres (n = 14) is planned; however, if recruitment rate indicates the need, the number of recruiting centres will be increased accordingly. The initial centres involved in the CSAW Study are known to the study team from previous collaboration in the United Kingdom Rotator Cuff Trial (UKUFF study; ISRCTN97804283). Their contribution and recruitment rates were reviewed in preparation for the CSAW Study protocol.

### Nonrandomised patients

Patients with subacromial pain who are eligible for trial participation but do not want to be randomised owing to their strong treatment preferences may still be included in a cohort substudy. With their consent, these patients will complete a questionnaire regarding their treatment preferences, reasons for not wanting to participate in the trial and a set of standard outcomes. This information may help with the analysis of the feasibility data by indicating if and why recruitment may be suboptimal or failing. It will also help meet Consolidated Standards of Reporting Trials requirements and the design and implementation of future, related studies. We anticipate recruiting up to 300 patients to this ‘preference-only’ part of the study. Questionnaires will be coordinated by the central study office in Oxford.

### Primary and secondary outcome measures

#### Primary outcome measure

The CSAW Study primary outcome measure is the OSS at 6 months postrandomisation. The OSS is a patient-based questionnaire and is a validated and effective measure of change over time [39].

#### Secondary outcome measures

The following are the secondary outcome measures:

- OSS at 12 months postrandomisation
- Constant-Murley shoulder score: assesses shoulder function and range of motion and is commonly used by health care practitioners [40]
- PainDETECT: a patient-based screening questionnaire used to predict the prevalence of neuropathic pain components that has been widely used in many areas of musculoskeletal care [41]
- Quantitative sensory testing: measures pain and pain thresholds (This is a modified form of the full quantitative testing and was validated by Gwilym *et al*. in 2009 [42].)
- Complications during and after the treatment.
- EQ-5D: a measure of general quality of life
- Health service use: measures the cost utilisation associated with the treatments
- Treatment expectations: Expectations will be assessed using two different scores. The first score was developed by Henn *et al*. [43] and is calculated using the Treatment Expectations Questionnaire. This questionnaire asks six general questions about pain and function. More shoulder-specific expectations will be predicted by asking patients to state how they expect to feel and function in 12 months’ time using the OSS.
- Patient satisfaction: Patient satisfaction will be assessed using the Oxford Satisfaction Index. This index includes transition questions developed and used recently by members of the study team to measure patients’ satisfaction after shoulder surgery [44].
- Anxiety and depression: The Hospital Anxiety and Depression Scale will be used to detect states of depression and anxiety in the setting of a hospital medical outpatient clinic [45].

The timing of all assessments is shown in Table 1 as a schedule of events. It is acknowledged that there are many outcome assessments, and questionnaire fatigue may be an issue. This will be closely monitored. Poor response rate to follow-up will be used as a marker of questionnaire fatigue.

**Table 1:** **Timetable of assessment** ^**a**^

**Assessment**	**Baseline**	**6 mo**	**1 yr**	**1 yr posttreatment (for waiting list breachers only)**
OSS (self-reported pain and function)	▲	▲	▲	▲
Constant-Murley shoulder score	▲	▲	▲	▲
QST and PainDETECT	▲	▲	▲	▲
EQ-5D (health economics)	▲	▲	▲	▲
Health service use		▲	▲	
Treatment expectations and patient satisfaction	▲	▲	▲	▲
Complications	N/A	▲	▲	▲
Patient response shift	N/A	N/A	▲	▲
Hospital Anxiety and Depression Scale	▲	▲	▲	▲

^a^N/A, Not applicable; OSS, Oxford Shoulder Score; Quantitative sensory testing. All information is postrandomisation, unless stated otherwise.

▲Outcome measure to be collected at this assessment.

### Study participants

Diagnosis of subacromial shoulder pain will be confirmed by a consultant surgeon. A clinical history and a clinical examination will be completed by the recruiting surgeons or members of their team. This will be done to ensure that the symptoms and pain course are indicators for arthroscopic surgery. Other possible shoulder diagnoses, such as frozen shoulder, full-thickness rotator cuff tears and instability, will be ruled out as much as clinical diagnosis allows.

#### Inclusion criteria

- Subacromial pain of at least 3 months’ duration (tendinopathy and partial tear only)
- Consultant’s clinical diagnosis of tendinopathic pain or partial thickness rotator cuff tear (using local pathways of diagnosis, which may include X-rays, magnetic resonance imaging (MRI) scans or ultrasounds)
- Eligible for arthroscopic surgery
- Completion of a conservative management programme previously, including both:
  - Physiotherapy that includes a remedial exercise regimen
  - At least one cortisone injection

#### Exclusion criteria

The participant may not enter the study if any of the following apply:

- Full-thickness tear of the rotator cuff tendons or calcific tendonitis evident on routine imaging
- Other shoulder pathology (non-impingement-related) identified on MRI scan or ultrasound
- Undergone any of the following surgeries on the affected shoulder:
  - ASAD
  - Cuff repair
  - Joint replacement
  - Surgery involving the glenohumeral joint (GHJ) in the past 3 years
- Rheumatoid arthritis or any other inflammatory disorder of the joints
- Symptomatic cervical spine pathology
- Previous septic arthritis in the shoulder only
- History of radiotherapy on same side as affected shoulder
- Patients who:
  - Are unlikely to be able to perform the required clinical assessment tasks
  - Have significant cognitive impairment or language issues
  - Are unable to provide consent for themselves
- Older than 75 years of age

### Study procedures

#### Recruitment organisation

Patients who would, in a routine care pathway, be considered for shoulder surgery for subacromial pain will be identified in outpatient clinics. Consultant orthopaedic surgeons and members of their teams will introduce the study to the patients and refer them to a research nurse and/or physiotherapist for further information. In all cases, the consultant orthopaedic surgeon will approve a patient’s eligibility for the study. Patients will be provided with written information about the study and asked to opt in. If their interest continues, they will be provided with further written information and arrangements will be made for a baseline appointment for assessment, consent and, in some situations, QRI conversation analysis audiotaping. The baseline assessment will be done as a separate appointment. These arrangements will be individualised for each centre. A reflection period for consent of at least 48 hours will be given following introduction of the study, but it is proposed that these appointments occur within 1 month of the initial approach. Consent will be obtained for any QRI-related audiotaping of appointments.

At the baseline appointment, patients will meet with a member of the CSAW Study research team again to discuss the process and to discuss the study further. The surgeon may also attend this visit to reiterate why the study is being conducted. Patients will be asked to sign an informed consent form, and baseline questions will be completed. Patients will be recruited over a 36-month period. Further details of the recruitment and follow-up processes can be found in Additional file 2.

#### Randomisation

After written informed consent is obtained and patient eligibility is confirmed, and immediately after the baseline appointment, patients will be randomised into the study by site staff authorised to perform telephone randomisations. Randomisations will be performed centrally by the Oxford Clinical Trials Research Unit within office hours (8 am to 5 pm), Mondays to Fridays, excluding public and/or bank holidays. The following information will be required at the randomisation call: participant details, research site details, name of caller, name of treating consultant, confirmation of eligibility, confirmation of written informed consent and its date, and stratification factors (see below).

Patients will be allocated a study number and will be randomised on a 1:1:1 basis to receive one of the three treatment options: ASAD surgery, AO or AMSR.

Randomisation will be performed using an automated, computer-generated minimisation system to ensure that treatment groups are well balanced for the following patient characteristics, the details of which will be required for the randomisation:

- Age (<40, 40 to 55 or 56+ years)
- Sex
- Baseline OSS (<19, 19 to 26, 27 to 33 or 34+)
- Centre

#### Surgery

Patients randomised to surgery will immediately be placed on the waiting list for surgery, with the expectation that surgery will be performed within 4 months. This reflects the average current surgical waiting time in the NHS. The surgical group to which the patient has been allocated will be concealed from the surgical team and revealed only on the day of planned surgery.

#### Active monitoring with specialist reassessment

Patients randomised to the AMSR group will receive a letter stating that a specialist reassessment appointment will be scheduled for a date 3 months hence. The reassessment will identify any patients who require adjustment to their management.

#### Informed consent

Patients will be asked to opt in to the study before receiving further information and asked to attend a baseline appointment. The opt-in form does not obligate the patient to participate in the study, but it will allow members of the CSAW Study team (both locally and centrally) to contact them further. Full consent will be obtained during the baseline visit by the participating surgeon or by qualified local centre study staff. The Principal Investigator at this centre will have overall responsibility for consenting patients, but can delegate the task to reliable members of the study team. Such delegation will be recorded on a Task and Responsibilities Log during centre initiation. Informed consent will be obtained according to Good Clinical Practice guidelines. Patients will be given sufficient time to accept or decline involvement. They will be free to withdraw from the study at any time without affecting their routine care.

Patients who complete the opt-in form but decline to be randomised may also be contacted in relation to the CSAW Cohort Study and the QRI. Satellite studies associated with the CSAW Study will have their own informed consent process. These are detailed later in the protocol.

#### Qualitative recruitment investigation

To characterise and understand the success or failure of the CSAW Study recruitment process, the QRI findings will be reviewed. These data will inform changes to aspects of the design, conduct and organisation of the trial and future surgical trials and to provide training which could potentially lead to improvements in recruitment. All findings and any suggested changes are fed back first to the CI and RCT staff involved in the TMG. Further details of the CSAW Study QRI process can be found in Additional file 1.

#### Study assessments

Participating surgeons will be asked to coordinate their waiting lists to ensure the CSAW Study patients are called for surgery in accordance with the study protocol. Prestudy questionnaires have been designed to screen sites for their ability to conform. Ideally, patients will complete their baseline assessments as close to the randomised treatment as possible (within 4 months). However, owing to the expected variability inside the 4-month window, and in the event of the unfortunate (but quite possible) breach of this 4-month window in a minority of patients, contingency measures have been incorporated to maintain design integrity. In the event that surgery cannot be performed within 4 months of randomisation, the same questions and assessments completed at baseline will be repeated. This assessment will be performed within the 2 weeks leading up to the patient’s operation (either on the day of surgery or at the preoperative assessment appointment). This will highlight any changes in the patient’s shoulder pain and function.

Follow-up measurements will be conducted postrandomisation. All patients will be followed up at 6 and 12 months postrandomisation, with patients in the AMSR arm having their specialist reassessment at 3 months postrandomisation. To capture the full effect of the intervention, patients who are on surgical waiting lists for longer than 4 months will have an additional 12-month posttreatment assessment. Each follow-up point will involve a clinical assessment, so patients will be asked to attend hospital for an appointment. Clinical assessments and questionnaires can be completed during these visits. The attendance of patients for an appointment will also ensure that the patients allocated to the nonoperative arm receive the specialist reassessment.

#### Compliance and loss to follow-up

We anticipate that 90% of patients will accept their allocated treatment (no immediate crossover), and therefore we have accounted for a dropout rate of 15% from the entire study over time. These estimates are based on assessments of our UKUFF study data. Compliance and withdrawals will be monitored as the CSAW Study progresses. The end of the study is set as the date of the last follow-up visit of the last patient.

### Study interventions

Patients randomised to surgery will remain unaware of which procedure they have undergone (ASAD or AO). The CSAW Study surgical procedures will mirror each other. Both operations will be performed through two arthroscopic keyholes. Standardised postoperative care and rehabilitation will be applied for both groups (ASAD and AO). If any unexpected pathology is encountered, such as full-thickness rotator cuff tear, frozen shoulder or arthritis, the patient will receive treatment for this pathology while under the same anaesthetic. In such a case, the participant will continue to be followed up for the study. Preoperative imaging of the shoulder (ultrasound and MRI scan) is known not to be fully accurate (inaccuracy rate of approximately 5%), and it is usual practice to advise patients of this. As a consequence, consent is routinely obtained in surgical settings for any additional treatment that may be required if an unexpected pathology or problem is encountered. The CSAW Study protocol dictates that when consent for the surgery is taken, surgeons will inform patients that they will be consented for shoulder arthroscopy, plus or minus an arthroscopic subacromial decompression, plus or minus a rotator cuff repair. This will ensure that patients are informed of the risks of surgery and potential eventualities. Further details of the individual interventions are given below.

#### Arthroscopic subacromial decompression

The procedure is performed with the patient under general anaesthesia. Skin incisions are made for the introduction of the arthroscope and required instruments. The procedure involves insertion of the arthroscope into the GHJ, where the joint surface is inspected along with the intraarticular portion of the long head of biceps and the joint surface of the rotator cuff tendons. Once this has been performed, the arthroscope is removed and inserted into the subacromial bursa, which lies outside the rotator cuff tendons and beneath the acromion process of the scapula. In the bursa, the acromion and superior surface of the rotator cuff are assessed to ensure that the coracoacromial ligament and the AC joint remain intact. The projecting undersurface of the distal part of the acromion is resected. The intervention is considered a well-established and well-documented procedure.

#### Arthroscopy only

The AO arm is the surgical comparison group. The procedure is performed with the patient under general anaesthesia. Patients will undergo a routine investigational arthroscopy of the joint and rotator cuff tendon. The operation will be performed in exactly the same manner as that in the ASAD group. Investigational arthroscopy has all the same essential operative components (and risks) of ASAD, but it does not involve surgical removal of any spurs or bursal tissue or release of the coracohumeral ligament. The procedure does involve the GHJ and the subacromial bursa being inspected and irrigated. Structures can be assessed for integrity and damage. The rotator cuff can be assessed for evidence of full-thickness tears. The synovium and lining of the shoulder can be assessed for evidence of capsulitis, arthritis or frozen shoulder. The time spent in the operating theatre will be similar to that for the ASAD group. These measures provide the AO group with the characteristics necessary to provide a reasonable comparison and account for the placebo effects of surgery.

#### Active monitoring with specialist reassessment

Patients will be advised that they will undergo active monitoring. They will attend a reassessment appointment 3 months after entering the study. At that appointment, they will be asked to complete questionnaires related to their shoulder pain and undergo a clinical assessment of the shoulder, including a record of any further conservative treatment. If, at the end of the 6-month assessment period, patients remain sufficiently symptomatic to require further intervention (based on clinical judgement), then additional treatment options will be discussed. It should be noted that the inclusion criteria state that all patients will have undergone conservative treatment (including physiotherapy and injection) before entering the trial. From an ethical standpoint, it is emphasised that it is quite within normal practice to have a period of active monitoring.

### Blinding

Blinding is performed for both patients and postintervention assessors in the surgical groups. For the AMSR group, blinding of the postintervention assessors only is appropriate and therefore will be applied. There will be a nominated unblinded person at each participating site. This person will be the main contact person for the patient and for the central study team in Oxford. Patients will be encouraged to contact this person for queries, if they have any complications and if they wish to pursue a different treatment option.

### Safety

There are no anticipated safety issues with the CSAW Study. Data on complications will be recorded as outcomes, and their severity and frequency will be assessed. Standard NRES safety reporting measures will be adhered to.

### CSAW Study complications

Expected complications in the CSAW Study are as follows:

1. Frozen shoulder
2. Joint and/or soft tissue infection
3. Further shoulder surgery

Local study teams that identify any complications will notify the CSAW Study office in Oxford. To maintain blinding, further information about the complications will be collected from the patients or the unblinded person at the corresponding site. The CSAW Study team in Oxford will coordinate collection of these data. Complications will be deemed serious if they meet the criteria listed below.

### Definition of serious adverse events

A serious adverse event (SAE) is any untoward medical occurrence that

- Results in death
- Is life-threatening
- *Note*: The term *life-threatening* in the definition of *serious* refers to an event in which the participant was at risk of death at the time of the event; it does not refer to an event which hypothetically might have caused death if it were more severe.
- Requires inpatient hospitalisation or prolongation of existing hospitalisation
- Results in persistent or significant disability and/or incapacity or is a congenital anomaly/birth defect
- Other important medical events

*Other events* refers to any event that may not result in death, is not life-threatening or does not require hospitalisation, but may be considered a serious adverse event when, based upon appropriate medical judgment, the event may jeopardise the participant’s health and may require medical or surgical intervention to prevent one of the outcomes listed above.

### Reporting procedures for unexpected serious adverse events

An unexpected serious adverse event (USAE) should be reported to the REC that gave a favourable opinion of the study when, in the opinion of the CI, the event is:

- Related (that is, it resulted from administration of any of the research procedures)
- ‘Unexpected (that is, the type of event is not listed in the protocol as an expected occurrence)

Reports of related SAEs and USAEs should be submitted within 15 days of the CI’s becoming aware of the event, using the NRES Report of Serious Adverse Event Form (available from: http://www.dt-toolkit.ac.uk/resourceindex/all.cfm).

### Statistics and analysis

#### Target sample size

A total sample size of 300 patients is planned. For a balanced three-group trial, this means that approximately 100 patients will be recruited per allocation group (ASAD, AO and AMSR).

#### Sample size calculation

The sample size calculation is based on the primary outcome measure, the OSS at 6 months postrandomisation.

Currently, there is no established minimal clinically important difference (MCID) for the OSS. In the absence of this, an approximation to the MCID can be based upon the change in the score between pre- and posttreatment. One-half of the standard deviation (SD) of this change could be viewed as an important change. In previous studies, the SD of the change in OSS has been estimated to be 9.0, giving a target difference of 4.5.

Using a two-sided *t*-test, 90% power to detect a difference in OSS of 4.5 (SD 9.0), with a two-sided 5% level of significance (α), a sample size of 85 participants is needed in each group. Accounting for dropouts and loss to follow-up of up to 15%, 100 participants are required in each group. Therefore, we aim to recruit a total of 300 participants (100 per treatment group) into the CSAW RCT. No adjustment was made for multiple comparisons.

#### Recruitment rate and access to the National Health Service

A total of 300 participants need to be recruited over 36 months. For 14 centres, this would mean an average recruitment of 21 to 22 patients per centre over 3 years (or approximately 7 patients per centre per year). As one-third (approximately 7) of these will be allocated to the AMSR group, the number of patients recruited for a surgical procedure will be around 14. When considering the availability of operating theatre time for this procedure, 14 operations are considered entirely manageable within 3 years. The addition of any further centres to the trial would decrease the average number of operations per centre required for the trial.

#### Analysis of endpoints

##### General considerations

The statistical analysis of the CSAW Study is the responsibility of the trial statistician. A full statistical analysis plan will be written by the trial statistician and agreed upon by the independent Data Monitoring Committee (DMC) before any analyses are undertaken.

Baseline data will be compared to determine whether there are any clinically important differences between treatment groups that have occurred by chance. It is anticipated that STATA statistical software (StataCorp, College Station, TX, USA), or another validated statistical software package such as SAS (SAS Institute, Cary, NC, USA) or R (versions will be recorded in the Statistical Report), will be used in the analysis.

All statistical testing will be performed at the two-sided 5% significance level, and 95% confidence intervals will be presented where appropriate. For all analyses, the appropriate model assumptions will be verified, and alternative methods will be used if more appropriate.

Prior to any analysis, any missing data pattern will be investigated and reasons for missing data obtained and summarised where possible. The primary analysis will be conducted on the modified intention-to-treat analysis, which includes all participants with nonmissing outcome data, unless there is clear evidence that its underlying assumption is inappropriate. Sensitivity analysis will be performed to assess the robustness of the results by imputing missing data using multiple imputation under both missing at random and missing not at random assumptions.

##### Frequency of analysis

A DMC will be set up to independently review the data on safety, protocol adherence and recruitment. Interim reports will be presented to the DMC in strict confidence at a minimum of yearly intervals. As no formal stopping rules have been specified for this trial, no formal interim analyses are planned, and hence no statistical testing will take place until the final analysis. The final analysis will take place after all participants have completed their follow-up and sufficient time has been allowed for data entry and validation.

##### Primary endpoint analysis

The primary outcome measure is the OSS measured at 6 months postrandomisation. The treatment groups are ASAD, AO and AMSR.

Multivariate linear regression analysis (analysis of covariance (ANCOVA)) will be used to examine the effect of the randomised intervention on the OSS at 6 months postrandomisation using three separate two-way comparisons (that is, ASAD vs. AMSR, ASAD vs. AO, and AO vs. AMSR). The model will be adjusted for (continuous) baseline OSS, sex, age and other relevant patient characteristics if appropriate. The minimisation factors (excluding centre) which are of clinical importance will be adjusted for in the model, regardless of statistical significance. Transformation of skewed variables will be considered, as will inclusion of polynomial terms if appropriate.

In additional secondary analyses, we will investigate the effect on the treatment estimates when long waiting times before surgery are taken into account. Participants with long waiting times before surgery may not yet have reached their optimal point of recovery when assessed at 6 months postrandomisation, and their data may underestimate the true treatment effect of their intervention. Analyses will include substituting the baseline and 6 months postrandomisation data for data collected at the presurgery and six months postsurgery assessments, respectively, for participants with a delay longer than 16 weeks before surgery. For participants with a long delay before surgery, the 6 months postsurgery assessments will coincide approximately with their 12 months postrandomisation assessment.

##### Secondary endpoint analyses

Linear regression analysis (ANCOVA) will be used to examine the effect of the randomised intervention on the OSS at 12 months postrandomisation, using three two-way comparisons. The model will be adjusted for appropriate baseline measurements. In addition, a repeated-measures multilevel model will be used, including repeated measures of the OSS at 6 and 12 months (level 1) nested within participants (level 2). Adjustments will be made for the continuous baseline OSS, other relevant minimisation factors and baseline measures as appropriate. Additional supplementary analyses will take into account long waiting times before surgery. As described for the primary analysis, analyses will include substituting the baseline and 12 months postrandomisation data for data collected presurgery and at approximately 12 months postsurgery, respectively, for participants with a delay longer than 16 weeks before surgery. In sensitivity analyses, we will look at the impact of a patient response shift on perceived disability by comparing each patient’s preoperative self-evaluation of his or her own disability (baseline OSS) with a 12-month evaluation of perceived preintervention state (the ‘thentest’) according to the method of Razmjou *et al*. [46]. This analysis will give an indication of whether patients with subacromial pain underrate or overrate their preintervention disability according to their outcome. The response shift will be presented descriptively using medians, interquartile ranges and ranges overall, as well as by treatment arm. The effect of the response shift and perceived disability will be investigated by refitting the above-described linear regression model for the OSS at 12 months postrandomisation, adjusting for continuous retrospective baseline OSS (thentest), as well as other relevant minimisation factors and baseline measures. Differences in the treatment effects using this approach and the previously described model for the analysis of the 12 months postrandomisation OSS will be highlighted.

The Constant-Murley shoulder score, PainDETECT, quantitative sensory test, Hospital Anxiety and Depression Scale and EQ-5D (index score and Visual Analogue Scale) measures will be scored according to their respective scoring manuals. Linear regression analysis (ANCOVA) will be used to analyse the 6- and 12-month outcomes, respectively, and a repeated-measures multilevel model will be fit (see above). The analysis will be adjusted for the relevant minimisation factors and baseline measures as appropriate.

### Complications

Complications between the trial arms will be compared in terms of frequency, seriousness and timing. To coincide with the primary endpoint, complications occurring within the initial 6 months from randomisation will be summarised first; complications reported over the entire duration of the trial will be reported separately. Logistic regression analysis with covariate adjustment will be carried out to test the difference in the proportion of participants with at least one complication, given sufficient events.

### Treatment expectations and patient satisfaction

Treatment expectations will be summarised descriptively using medians, interquartile ranges and ranges overall, as well as by treatment arm. Patient satisfaction with treatment will be summarised descriptively by treatment arm for the 12-month time point. Cross-tabulations will be generated to relate treatment expectations to treatment satisfaction, and χ^2^ tests will be used to assess the significance of the association.

### Cost-effectiveness

The trial health economist will analyse the cost-effectiveness of the three treatment arms. A specific plan of analysis will be written for the health economics analysis.

### Qualitative recruitment investigation

Thematic analysis will be used to identify common and emergent themes in the interview data by employing constant comparison techniques until no new themes emerge. Throughout the analysis, the perspectives of the recruiters will be paramount. Content analysis will be used to describe the terminology used by the recruiters and compare this with written study information. Discrepancies and areas of controversy will be identified and explored. Conversation analysis will be used to investigate the delivery of information during the recruitment appointments, with a particular focus on the interaction between recruiter and patient (for example, analysis of patient requests for clarification or places in the conversation where pauses or other utterances disrupt the smooth flow of interaction).

There will be frequent assessments of recruitment rates—both randomisation and rates of compliance with assigned treatment. These will be calculated for each of the centres in which the QRI is running and across the trial as a whole.

### Ethics and participant confidentiality

The study has been designed under the ethical supervision of an academic ethicist (J.S.). The CSAW Study does not include a true sham surgery treatment arm, but it has comparisons that account for any placebo effects expected with surgical procedures and will also allow detailed assessment of the mechanism of effect. We are satisfied that all potential ethical concerns have been explored and discussed and that the optimum route has been taken, with patient welfare foremost but with study validity taken into account.

The study staff will ensure that the participants’ anonymity is maintained. The participants will be identified only by initials and a participant identification number on the Case Report Form and in any electronic database. All documents will be stored securely and will be accessible only by study staff and authorised personnel. Transcripts of all audio recordings will be anonymised, and audio recordings will be labelled and stored with a patient code to protect participant anonymity. The study will comply with the United Kingdom’s Data Protection Act 1998, which requires data to be anonymised as soon as it is practical to do so.

A DMC will be set up to independently review data on safety, protocol adherence and recruitment in the CSAW trial. Interim reports will be presented to the DMC in strict confidence in at least yearly intervals. This committee, in light of the interim data and any advice and evidence they wish to request, will if necessary report to the Trial Steering Committee (TSC) if there are any concerns regarding the safety of the trial treatments. Although there are no formal stopping rules specified for the CSAW trial, the CSAW trial team, together with the DMC, will closely examine the recruitment of participants into the trial. If recruitment is low compared with the specified targets, then reasons for low recruitment will need to be explored and necessary steps will need to be taken. Should it become clear that potential participants refuse to consent into the trial because of an unwillingness to be randomised to the nontreatment trial arm, recruitment and consent strategies will need to be verified and if this should not result in improved recruitment, then the AMSR trial arm may have to be dropped.

### Data handling and record-keeping

Data management will be performed via a web-based trial database (OpenClinica: http://www.openclinica.org/) and managed by the Oncology Clinical Trials Office (OCTO). OpenClinica is a dedicated and validated clinical trials database designed for remote electronic data capture.

The CI will act as data custodian for the trial. A guide explaining how to use OpenClinica will be provided to every site. The CSAW Trial Coordinator and OCTO monitors will have an overview of all entered data.

The participants will be identified by a study-specific participant number and/or code in any database. The name and any other identifying detail will not be included in any study data electronic file. Any patient-related data transferred between the main study office and participating sites will be identifiable only with each patient’s unique study number. If more identifiable information is required, secure measures such as registered post, courier or nhs.net e-mail accounts will be used. For quality control reasons, monitoring of site files and data collection forms may be initiated by the main study team.

Audio recordings will be stored on password-protected devices provided by the University of Bristol and transferred by recruiting centres on encrypted mobile memory drives (pen drives). Codes for storing and labelling patient and recruiter audio data will be generated by researchers at the University of Bristol once written informed consent has been received from the participants. Signed copies of informed consent forms will be kept in a locked filing cabinet by researchers at the University of Bristol. The CI will be held responsible for their safe keeping.

### Financing and insurance

The study is funded by Arthritis Research UK via a clinical studies grant. The total grant is £332,389.76. The University of Oxford has arrangements in place to provide for harm arising from participation in the study, for which the university is the research sponsor. NHS indemnity operates in respect of the clinical treatment which is provided. The University of Oxford has arrangements in place to provide for nonnegligent harm arising from participation in the study, for which the University of Oxford is the research sponsor.

### Publication policy

The trial investigators will be involved in reviewing drafts of the manuscripts, abstracts, press releases and any other publications arising from the study. Authors will acknowledge that the study was funded by Arthritis Research UK. Authorship will be determined in accordance with the International Committee of Medical Journal Editors guidelines, and other contributors will be acknowledged.

### Satellite studies

Separate protocols have been written that detail the related substudies. Any future satellite studies will have their own protocols and their own consent forms.

#### CSAW neuroimaging observational study

Functional magnetic resonance imaging (fMRI) studies of the brain will be included in the study to investigate the neural correlates of the ASAD and compare them with nontherapeutic diagnostic arthroscopy (the AO group). fMRI is an objective tool used to assess underlying biological processes responsible for pain. fMRI uses the same techniques as clinical MRI examinations. It is noninvasive and safe, and it allows for repetitive measurements in the same individual. fMRI has an advantage over other imaging techniques in that it avoids exposure to ionising radiation or radioactive tracers. fMRI has been demonstrated to be an objective and sensitive tool for assessment of the effect of therapeutic interventions on pain in patients with chronic pain. This is applicable only to patients recruited from Nuffield Orthopaedic Centre, Oxford University Hospitals NHS Trust, Oxford, UK.

#### CSAW tissue sample study

An analysis of tissue excised and routinely discarded during ASAD will also be conducted. This includes bursa, coracoacromial ligament and acromion bone tissue. This proposed study will involve only one arm of the study (ASAD) and not the other two (AO and AMSR groups).

Small rotator cuff tears elicit a significant inflammatory response which decreases as tear size increases [47]; however, relatively little is known about changes in the neuronal signalling that occur in patients with subacromial pain and rotator cuff degeneration. It has been shown that substance P levels in the subacromial bursa are significantly higher in patients with painful rotator cuff tears [48], but the way in which inflammation and painful tendon degeneration are affected by neuronal signalling is poorly understood.

Our aim is to determine which neuronal and cell signalling changes occur during tendon degeneration, thus furthering understanding of the key molecular mechanisms involved in this process. Tissue from the subacromial bursal area from patients in the ASAD group is removed routinely as part of their surgical procedure. Our aim is to analyse this tissue to investigate the relationship between variations in peripheral neuronal innervation, nociceptor density, nerve growth factors and clinical presentation with preoperative pain levels and with outcome following surgery. A better understanding of the key molecular processes involved in tendon degeneration and shoulder pain also has the potential of leading to novel therapeutics.

## Discussion

As an innovative design and one that involves a placebo surgical intervention, this trial protocol has several aspects that warrant further mention.

### Intervention content

The intervention content for each of the groups was carefully selected. The content of the ASAD was chosen to represent what is most likely to be current practice for this operation. Some level of consensus was obtained by polling specialist society members (British Elbow & Shoulder Society), but no formal confirmation of best practice was obtained. It is suggested future trials might formally define appropriate content for the best practice surgical intervention. An AO intervention was deemed ethical, as it is often considered an appropriate form of surgical treatment for this condition, albeit mainly for diagnostic and evaluation purposes. Likewise, a nonsurgical arm was considered ethical, as the inclusion criteria required all patients to have undergone previous conservative management. A nontreatment period is often advocated at this point in the pathway. Terminology was important for the AMSR group. A group described as having ‘no treatment’ would have been considered to pose a threat to successful recruitment.

### Identification of the critical surgical element

Careful consideration was given to ensuring that the critical surgical element was identified and omitted for the placebo intervention group (AO). Whilst this was relatively straightforward for the CSAW Study (removal of bone), it may not be quite so obvious for other surgical interventions which may have a more cumulative effect of the surgery.

### Threats to recruitment

There are several aspects which need careful management to avoid the potential for poor recruitment. Patients might perceive that two of the three options in randomisation offer poor treatment alternatives. This could result in a high rate of refusal to participate. Careful wording and clear demonstration of the uncertainty for all three intervention options was required. The involvement and inclusion of an experienced qualitative research team provides significant benefit by exploring and addressing additional threats to recruitment. A further threat to recruitment could have been lack of surgeon equipoise. Again, the qualitative work and repeat demonstration of the uncertainty for practice, as well as its efficacy, can help dispel worries and concerns among the surgical investigators.

### Crossover between groups

There is a concern that patients might cross over treatment to surgery, especially among those in the active monitoring arm. Clear procedures and thresholds for clinical evaluation and crossover have been instigated to prevent this. Whilst treatment cannot be withheld on ethical grounds, patients are not actively encouraged to explore alternative treatment options throughout the study. The crossover rates of the control group to surgery will also be reviewed, and the trial will be reconfigured if needed.

### Waiting list effects

It is rare that patients can undergo surgery after randomisation without any delay, whereas those in the AMSR group begin their intervention immediately. Thus, waiting list effects can play a part in the study design and can introduce imbalance for follow-up times between groups. To compensate, additional follow-up assessments, referenced from surgery, have been introduced for patients allocated to surgery. Patients waiting for longer than 4 months for their surgery after randomisation are termed *breachers* and given additional follow-up appointments. The timing of these appointments ensures their assessment is not done at a time that conflicts with any healing profile (that is, too early after surgery) and is aligned to ensure appropriate comparison with other patients. Note, all patients will undergo the standard, scheduled 6- and 12-month postrandomisation follow-up assessments, regardless of time to surgery.

### Compliance and loss to follow-up

As the study follow-up (from baseline to primary endpoint) is only 6 months in duration, a very high level of follow-up is anticipated based upon previous experience. The number of assessments is not onerous for the patients and is spread out over 12 months. All attempts will be made to make these appointments as convenient for the patients as possible. The content of the assessment is also not burdensome, with predominantly standard clinical measures being taken and examinations being done. The assessments involve no discomfort for the patient over and above the routine clinical shoulder examinations. Although we do not expect any problems with compliance, this will be monitored throughout the trial. Patients who do not return follow-up questionnaires will receive reminder telephone calls. All possible means to minimise the amount of missing data will be employed. Refusal to participate and refusal to continue with allocated treatment are considered to pose the biggest threats, particularly the active monitoring allocation. Both of these are dealt with by the contingency plan to convert the trial to a two-arm trial upon completion of the early review stage.

### Interpretation

The importance of highlighting potential outcome and the clinical implications has been shown by the development of this trial. The complexities of the design mean that interpretation is not straightforward. If surgery (ASAD) were shown to be no more effective than AMSR, then a question would hang over the continued use of surgery for this condition in the NHS and in a wider context. Conversely, if ASAD (or shoulder arthroscopy in general) is shown to be an effective treatment, then the current enthusiasm for the procedure should be encouraged and similar increased availability within the health care system should be entertained. If ASAD is more effective than AO, then it will have been confirmed that it is necessary to remove an acromial spur during arthroscopy. As a comparison of ASAD with bursectomy alone has already been undertaken (showing no difference at 1 year), the value of repeating this is not apparent [27]. The difference in surgical morbidity and cost is not likely to be significant between ASAD and bursectomy alone. Clearly, there are health care economic implications, and a full economic costing and cost utility analysis will be performed for both types of arthroscopy.

## Trial status

The CSAW trial commenced in July 2012 and is ongoing. Recruitment was originally scheduled to end in June 2014. Initial slow recruitment, mainly due to governance issues and time taken to include and activate additional recruiting centres, meant that recruitment targets were not going to be met. An extension to the trial was recommended by the TSC and DMC, which was agreed upon by the funders. Completion of recruitment is imminent.

## Additional files

Additional file 1:
**Qualitative recruitment investigation.**
Additional file 2:
**Study flowchart.**

## Abbreviations

AC: Acromioclavicular
AMSR: Active monitoring with specialist reassessment
ANCOVA: Analysis of covariance
AO: Arthroscopy only
ASAD: Arthroscopic subacromial decompression
CI: Chief Investigator
CSAW: Can Shoulder Arthroscopy Work?
DMC: Data Monitoring Committee
fMRI: Functional magnetic resonance imaging
GHJ: Glenohumeral joint
MCID: Minimal clinically important difference
MRI: Magnetic resonance imaging
NHS: UK National Health Service
NRES: National Research Ethics Service
OA: Osteoarthritis
OCTO: Oncology Clinical Trials Office
OSS: Oxford Shoulder Score
PTT: Partial thickness tear
QRI: Qualitative research investigation
QST: Quantitative sensory testing
RCT: Randomised controlled trial
REC: Research Ethics Committee
SAD: Subacromial decompression
SAE: Serious adverse event
SD: Standard deviation
TMG: Trial Management Group
TSC: Trial Steering Committee
UKUFF: United Kingdom Rotator Cuff Trial
USAE: Unexpected serious adverse event
