# Supplementary material for: The CSAW Study (Can Shoulder Arthroscopy Work?) – a placebo-controlled surgical intervention trial assessing the clinical and cost effectiveness of arthroscopic subacromial decompression for shoulder pain: study protocol for a randomised controlled trial
Source: Trials. 2015 May 9;16:210. doi: 10.1186/s13063-015-0725-y (PMC4443660; doi:10.1186/s13063-015-0725-y)
Supplement: Additional file 1: — Qualitative recruitment investigation. [file 13063_2015_725_MOESM1_ESM.docx]

# Additional file 1: QUALITATIVE RECRUITMENT INVESTIGATION (QRI)

**1. Phase I: Understanding recruitment**

The aim of Phase I is to understand the recruitment process as it occurs. There are several distinct parts that can provide information about recruitment as it happens, and to identify and investigate the sources of recruitment difficulties.

**1.1** **Patient pathway through eligibility and recruitment**

A comprehensive process to log potential RCT participants through screening and eligibility phases will be put in place in order to ensure compliance with the trial protocol and to monitor recruitment. The main trial team will request all recruiting centres to provide a flow chart of the anticipated recruitment pathway that maps the patient’s journey beginning from the point of diagnosis to making a decision about participation in the RCT. Logs and flow charts will be assessed for complexity and compliance with the protocol and variation between centres. In particular, the logs will give an indication of the numbers of eligible patients and particular points where they are ‘lost’ from the RCT. They will also indicate levels of equipoise – as evidenced by the numbers rejecting participation in the RCT and the selection of particular treatments. Flow charts will indicate the degree of complexity of participation and variability between centres.

**1.2** **In-depth interviews**

Consent will be sought to audio-record in-depth, semi-structured interviews with the following persons involved in the CSAW trial:

1. Members of the TMG, including the CI and those most closely involved in the design, management, leadership and coordination of the trial
2. Clinical and recruitment staff across the range of clinical centres involved in the RCT
3. Participants eligible for recruitment to the RCT, including those who accept or reject randomisation

Interview schedules will be used to ensure similar areas are covered in each interview, based on those used in previous studies (refer to recruiter and patient interview schedules. Participants will also be encouraged to express their own views about the RCT and any recruitment difficulties. Participants in group (a) will be asked about the background, development and purpose of the RCT, including their knowledge of the evidence and equipoise; their role in the trial and recruitment, including their expectation of the pathway through eligibility and recruitment. They will also be asked to provide a short summary of the RCT for the interviewer, as if s/he were a patient. Informants in group (b) who directly recruit to the trial will also be asked questions about their knowledge of the evidence and personal views about equipoise; the recruitment pathway, how they feel the protocol fits their clinical setting and any adjustments they think are needed. They will also be asked how they explain the RCT and the interventions and controls to patients and the randomisation process, and will be asked to audio-record their appointments with patients. Informants in group (c) will include patients who agree to randomisation and accept or refuse the allocation, or refuse randomisation and choose their treatment. They will be asked about their understanding of the CSAW trial, their experience of recruitment and their reflections on the patient information sheet.

Informants will be purposively sampled. It is expected that the CI and most of the TMG will be interviewed. It will also be necessary to sample clinicians and recruiters. Initially, those having attempted recruitment will be selected, followed by further theoretical sampling based on emerging findings – for example the need to recruit clinicians from a particular specialty or centre. Interview recordings may be transcribed verbatim whole or in selected parts, as necessary to conduct comprehensive analysis. Transcripts will be analysed thematically by the QRI researcher, using techniques of constant comparison and case-study approaches. This will involve detailed coding, and then comparing emerging themes and codes within transcripts and across the dataset looking for shared or disparate views among TMG members, specialist clinicians and recruiters, and within or between centres or specialties. The coding will be carried out using qualitative data analysis software such as NVivo. The initial coding will be cross-checked by another researcher and discussed with the QRI PI, with inconsistencies resolved by discussion. Detailed descriptive accounts of the themes and cases will then be produced by the QRI researcher. Interviews and meetings will provide data about the evidence underlying the RCT, including the importance of the question and the commitment of staff to it, as well as individual clinical equipoise; the application of the protocol in clinical centres and any logistical issues; and suggestions about reasons for recruitment difficulties and potential solutions from those working closely with the RCT.

### 1.3 Observations of investigator meetings

It is likely in the early stages of the RCT that the CI, TMG and clinical investigators will meet several times during the first 6 months. Members of the QRI team will ask to observe these meetings and if appropriate seek permission to audio-record the discussion. The aim will be to gather further information about specific issues that may have a bearing on recruitment. If required, consent for audio-recording of any meetings will be requested and agreed in advance with CI and TMG members. The QRI researchers will discuss the agenda with the CI, with the aim of fostering discussion particularly about issues of eligibility and equipoise. The meetings will also be a forum to discuss the findings of the QRI, and to deliver training or advice about recruitment.

**1.4 Audio-recording of recruitment appointments**

The importance of audio recording discussions about RCT recruitment will be emphasised to the CI and TMG, and RCT-specific methods of communicating this with recruiters will be explored. It will be emphasised that the feedback to them will be confidential and positive (not critical). The CI and TMG will be asked to discuss this with recruiters and attempt to identify a ‘recruitment appointment’ suitable for recording.

The QRI team will work with the CI/TMG to identify centres where audio-recording of recruitment appointments would be most appropriate and feasible. These will be based on the existing screening log information, initially focusing on centres that attempt recruitment; and later driven by theoretical sampling following data analysis. One main point of contact (usually the lead research nurse) will be identified per centre and digital audio-recorders will be provided; the number of recorders required for the RCT will depend on the number of actively recruiting staff in the centre and the logistics and geographic location of recruiters. Recruitment staff will be requested to audio-record all appointments where they provide information to patients and attempt to recruit them to the RCT. Documents explaining the ethical requirements of audio-recording of patient appointments (Patient and Researcher Information Sheets and consent forms for audio-recording) are included as appendices.

In addition the QRI team will provide each participating centre a ‘Recruiter Pack’ with detailed guidance on the process of obtaining informed consent, the operation of digital recorders, how to record, name and transfer audio-files and documents to ensure information from the QRI is secure and confidential.

Audio-recordings of appointments will be analysed as described above for interviews, with the addition of some of the techniques of focussed CA – conversation analysis – pioneered in previous studies. CA techniques will be used to identify and document aspects of informed consent and information provision that is unclear, disrupted or hinders recruitment. Recordings will be listened to by the QRI researcher and notes made about the content of the appointment, including the basic content covered, the order of presentation of RCT arms and other treatment options, time spent on interventions and controls, and time spent describing both the RCT design and the randomisation process. An assessment will be made as to whether the appointment is recruiter or participant-led, and also the degree to which there is evidence that the participant has understood the key issues of equipoise, randomisation, participation in the RCT, the option to choose their treatment, and the option to withdraw from research at any time.

The QRI researcher will document these details and provide an account for the QRI PI. When at least three recordings have been analysed, the QRI researcher and PI will decide what confidential feedback will be given to the recruiter. Issues to be fed back to the RCT CI/TMG, or to be used anonymously in training programmes will be discussed and defined. These data will form the basis for feedback to individuals and to determine the content of its information, and training programmes to be initiated in Phase II.

**2. Phase II: Feedback to CI/TMG and plan of action**

The QRI researcher team will present a summary of anonymised findings emerging from Phase 1 to the RCT CI and TMG, identifying any aspects of the RCT design and conduct that could be hindering recruitment with the supporting evidence. There are likely to be several meetings during the feasibility phase of the study to present these findings and discuss a plan of action to try to improve recruitment, if this proves necessary. The plan will be agreed by the RCT CI/TMG and QRI PI and team. No activities will be undertaken by the QRI team without the prior approval of, and collaboration with, the RCT CI and TMG. The degree of involvement by the CI/TMG will be at their discretion. However, it is likely that the activities in the plan will need leadership from the CI/TMG as well as the QRI team if they are to be acceptable to the RCT team and thus effective.

The plan for the RCT will be focussed on the issues emerging from the ethnographic investigation (QRI) of the RCT and thus based on the details of the RCT and how it has been applied in clinical centres. It is likely that some aspects will be generic, such as difficulties explaining randomisation. The plan is likely to include some or all of: reconsideration of study information, advice about presenting the study, discussions about equipoise or evidence, issues with patient pathways and logistical issues. These may be addressed by a new patient information sheet, additional documents or training for recruiters.

**2.1 Evaluation of the Impact of the Plan**

Numbers of eligible patients, and the percentages of these that are approached about the RCT, consent to be randomised and immediately accept or reject the allocation will be assessed before the plan of action is implemented, and regularly afterwards to check whether rates are improving. Interview with recruiters will ask about the acceptability of the QRI and any changes that occur.

**2.2 Staff training and feedback**

Feedback and training will be focused on eliminating the reasons for inefficient recruitment uncovered by the qualitative research. Findings will be fed back to the recruiters individually and in staff training sessions. The researcher will continue to listen to recorded interviews and provide support, feedback and training sessions until recruitment rates improve and remain reasonably constant, or it is found that improvement is not possible.

### 3. Consent Procedures

All participants will be asked to provide informed consent prior to taking part in any aspect of the QRI. Interviews will be conducted at a time suitable for TMG members, recruitment staff and eligible patients and permission will be sought from participants to audio-record discussions. All data collected by will be used only for the purposes of improving levels of informed consent and acceptance of randomised treatment within CSAW. Findings from the QRI may be published in peer-reviewed journal articles as agreed by the CSAW CI/TMG and the PI of the CSAW QRI.

**3.1 Interviews with recruiters**

### Recruiters will be provided with an information sheet and asked to provide written consent to be interviewed and for their appointments with potential CSAW patients to be audio-recorded. They will be able to withdraw this consent at any time.

### 3.2 Audio-recording recruitment appointments - patient consent

The aim is to collect recordings of consultations in which treatment options are discussed with patients who either accept or decline randomisation into the CSAW study. Information about the audio-recording of discussions and invitation to participate in an interview is included in the revised version of the CSAW Patient Information Sheet If patients are willing to participate in the QRI, they will be asked to provide written consent to confirm they have read the project information sheet and consent to the audio-recording of their treatment discussions. It will be made clear to patients discussions are being recorded for research and training purposes to improve communication about shoulder complaints. Patients will also be asked for permission to be contacted by a researcher from the University of Bristol, with a view to participating in a follow-up interview (by telephone or face-to-face) in which they will be asked about their understanding of the CSAW trial, provision of information and follow-up treatment decision. Interviews will take place at a convenient time and place, approximately one week after the patient has made a final decision about their treatment. Interviews will last approximately 30 minutes. Patients will be asked for verbal consent to audio-record the interview. It will be made clear to patients the audio-recording of consultations and participation in an in-depth interview is entirely voluntary and confidential, and will not affect their treatment.

**4. Time frame**

This QRI will start in the first year of the CSAW trial. It is anticipated that the audio-recording of patient consultations will begin as soon as ethical approval has been obtained. Recruiter interviews will begin as soon as is feasible. Timing of any implementation of training the training programme will be agreed by the CSAW Trial Management Group.
