# Supplementary material for: The CSAW Study (Can Shoulder Arthroscopy Work?) – a placebo-controlled surgical intervention trial assessing the clinical and cost effectiveness of arthroscopic subacromial decompression for shoulder pain: study protocol for a randomised controlled trial
Source: Trials. 2015 May 9;16:210. doi: 10.1186/s13063-015-0725-y (PMC4443660; doi:10.1186/s13063-015-0725-y)

Additional file 2: Study Flow Chart

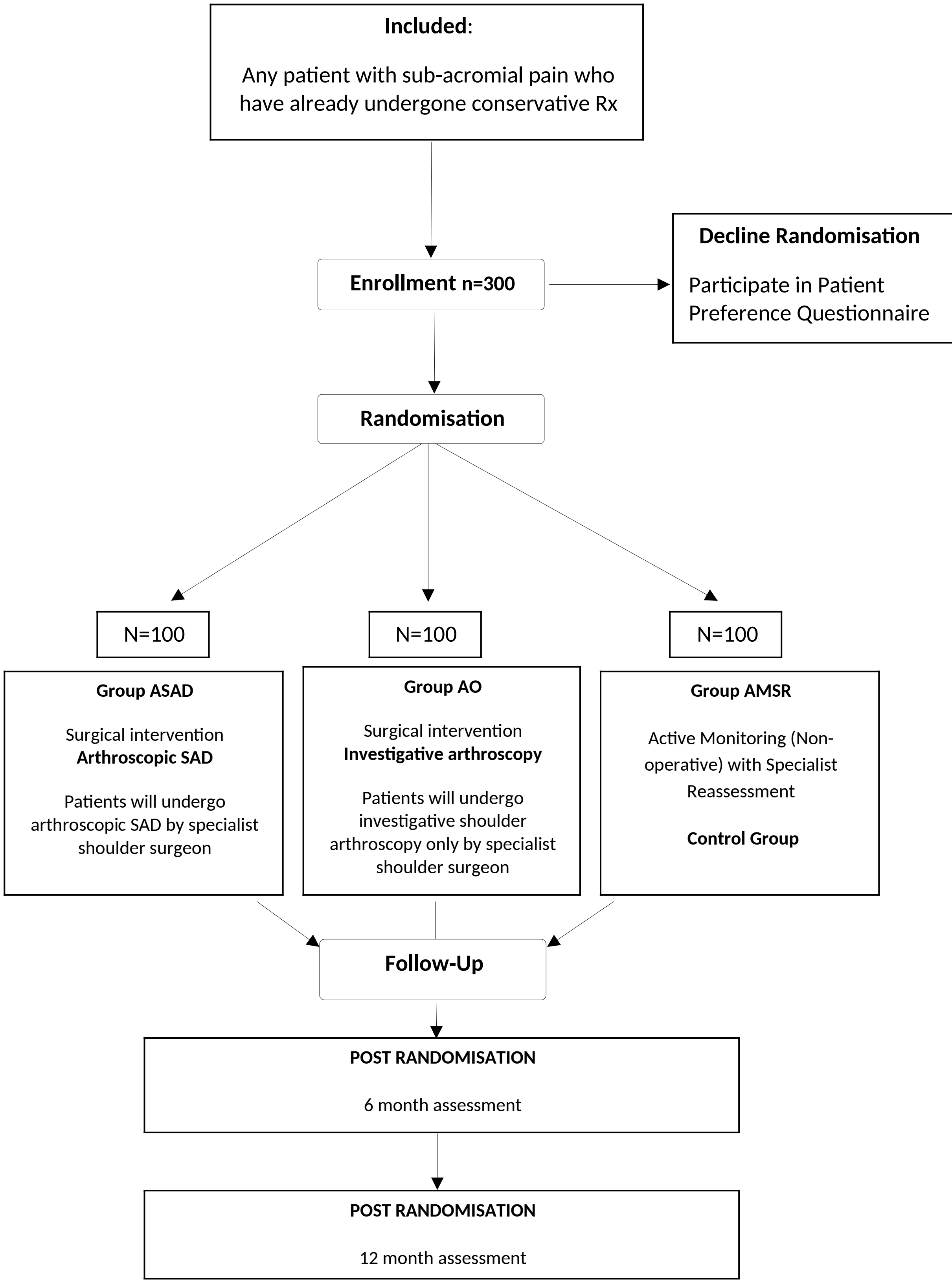

Supplement: Supplementary file 2 [file 13063_2015_725_MOESM2_ESM.docx]
